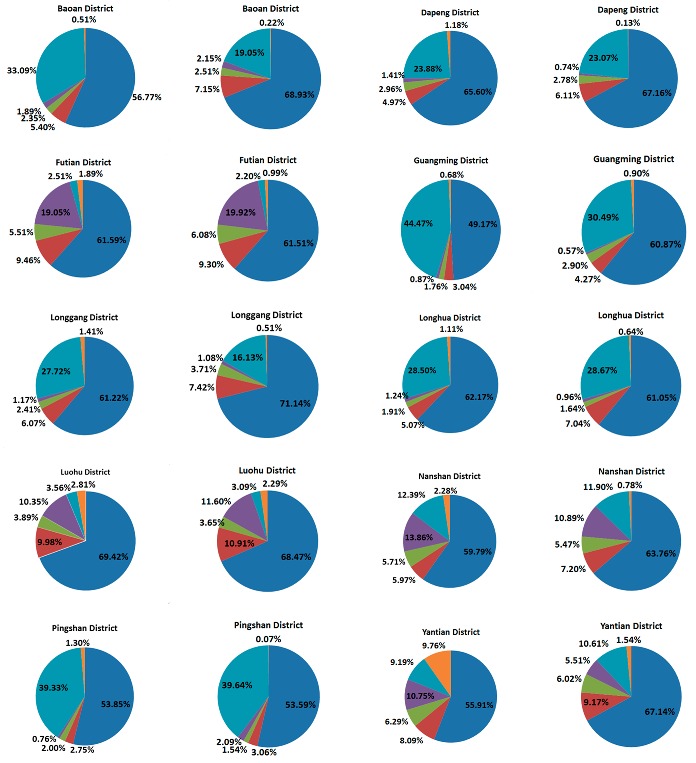# Building-Based Analysis of the Spatial Provision of Urban Parks in Shenzhen, China

**DOI:** 10.3390/ijerph14121521

**Published:** 2017-12-06

**Authors:** Wenxiu Gao, Qiang Lyu, Xiang Fan, Xiaochun Yang, Jiangtao Liu, Xirui Zhang

**Affiliations:** 1 School of Architecture and Urban Planning, Shenzhen University, Nanhai Ave 3688, Shenzhen 518060, China; wxgao@szu.edu.cn (W.G.); 2151140405@email.szu.edu.cn (Q.L.); 2140140608@email.szu.edu.cn (X.F.); 2 Shenzhen Key Laboratory for Optimizing Design of Built Environment, Shenzhen University, Nanhai Ave 3688, Shenzhen 518060, China; 3 Urban Planning, Land and Resources Commission of Shenzhen Municipality, Shenzhen 518034, China; jiangtao.liu@foxmail.com (J.L.); xrzhangchn@gmail.com (X.Z.)

**Keywords:** urban park, spatial provision, service area, service load, building floor area, building density map

## Abstract

Urban parks provide important environmental, social, and economic benefits to people and urban areas. The literature demonstrates that proximity to urban parks is one of the key factors influencing people’s willingness to use them. Therefore, the provision of urban parks near residential areas and workplaces is one of the key factors influencing quality of life. This study designed a solution based on the spatial association between urban parks and buildings where people live or work to identify whether people in different buildings have nearby urban parks available for their daily lives. A building density map based on building floor area (BFA) was used to illustrate the spatial distribution of urban parks and five indices were designed to measure the scales, service coverage and potential service loads of urban parks and reveal areas lacking urban park services in an acceptable walking distance. With such solution, we investigated the provision of urban parks in ten districts of Shenzhen in China, which has grown from several small villages to a megacity in only 30 years. The results indicate that the spatial provision of urban parks in Shenzhen is not sufficient since people in about 65% of the buildings cannot access urban parks by walking 10-min. The distribution and service coverage of the existing urban parks is not balanced at the district level. In some districts, the existing urban parks have good numbers of potential users and even have large service loads, while in some districts, the building densities surrounding the existing parks are quite low and at the same time there is no urban parks nearby some high-density areas.

## 1. Introduction

Urban parks are one of the indispensable green spaces of city life, and they play important roles in improving living quality, public health, social civilization and livable residential environments [1]. Many recent studies have intensively demonstrated the functions of urban parks on providing recreation opportunities [2], enhancing landscape aesthetic value [3], improving air quality and noise [4] as well as promoting the physical and mental health of people [5]. People who live close to an urban park have more opportunities for daily exercise and social interaction than those who live a bit farther away [6,7]. Studies show that daily contact with urban parks offers numerous benefits for physical health, mental health and personal well-being [8,9], and the accessibility to public parks for people with limited mobility has been stressed [7,10]. This is not only true for residential areas, as urban parks within walking-distance around workplaces, such as the Greenacre park at New York City (http://greenacrepark.org), also can encourage people to connect with nature and to have a short relax, especially during lunch time, which is welcomed by people working in the surrounding office buildings [1].

In earlier studies, the provision of urban public facilities was investigated by empirical surveying [11], and many applied census and social-economic data to evaluate the spatial and social provisions of urban public facilities (including urban parks) by taking the center points of census tracts as the locations of people [12,13,14,15,16,17]. The spatial associations between urban parks and their potential users are measured with the distances from the center points of tracts to the center points of urban parks. The results of such measure potentially suppose all of people living in different buildings at a tract have the same accessibility to an urban park. However, it is not always true, especially when the sizes of the census tracts vary greatly. For example, the largest tract in Luohu district of Shenzhen in China (called Tract 1) is 13 km^2^ and the smallest one (called Tract 2) is only 0.025 km^2^. The distance from the center point of Tract 1 to its edge is more than 2000 m and while the corresponding distance of Tract 2 is less than 900 m. A person in a building located at the center of Tract 1 needs to walk 2000 m more to an urban park outside Tract 1 than a person in a building at the edge of Tract 1, and there is no such big difference for people in Tract 2. By comparison, the spatial accessibilities to the urban park are much more varied for people in Tract 1 than people in Tract 2. Moreover, for a large-area urban park, the distance between its edges and its center point may also underestimate its spatial accessibilities since in practice a person already has accessed an urban park right after he crosses its entrance. Therefore, it is more accurate to measure the spatial accessibilities using the distance from individual buildings to the edge of urban parks, especially for large-area tracts and urban parks.

Building floor area (BFA) is used to describe the total usable floor space [18] and BFA per capita of residential buildings is used as an index in urban planning to estimate the basic conditions of integrated households of a specific area. Therefore, it is potentially supposed that the population able to be hold in residential buildings is proportional to BFA of this area. It is also accepted to assume that the population is proportional to BFA of business/commercial areas [19]. Since that, it is reasonable to apply BFA covered by the service area of an urban park to estimate the service coverage and potential service loads of the park. In addition, the usage information of buildings can assist to identify the distributions of urban parks at residential areas and workplace areas (such as commercial and industrial areas).

This study aimed to apply the above information of buildings to precisely evaluate the spatial provision of urban parks in a city. The basic idea is to take the spatial distribution of BFA to simulate the distribution of population to investigate the spatial associations between urban parks and their potential users. Based on such spatial associations, a BFA-based building density map was used to display the spatial distribution of urban parks and five indices were designed to measure the scales, service coverage and potential service loads of urban parks in an acceptable walking distance. These indices can describe the service capacities of urban parks from various points of view. Moreover, the areas lacking urban park services can also be identified to be alternative areas for new parks in the future.

Shenzhen is located at the south of Guangdong Province in China and is a significant test case for the urbanization of China. It has developed from several small villages with a population of 332,900 in 1980 into a megacity with a population of 10,778,900 in 2014 [20]. Rapid population growth may place great pressure on the sustainable development of urban areas because of crowding, environmental degradation and other impediments to productivity associated with rapid urbanization [21,22]. In order to protect the ecological environment, the Government of Shenzhen has taken measures to protect the natural environment and built urban parks and natural parks since 2004, and released regulations on developing urban parks with an objective of becoming an “urban park city” [23]. There are two kinds of urban parks in Shenzhen, city parks and community parks, which are built for providing services to people in their daily lives. Taking Shenzhen as a case, this study aimed to explore the spatial provision of city parks and community parks based on the idea discussed above.

Section 2 states the basic socio-economic contexts of the study area and the contents of data used in this study. Especially, in order to prove the feasibility of using information on buildings in such a study, a further analysis is added in Section 2.2.3. Section 3 proposes the methodology including BFA-based building density maps and the five indices described above. The results, discussion and conclusion are stated in the subsequent three sections.

The results of this study indicate that the spatial provision of urban parks in Shenzhen is insufficient and unbalanced. Less than 40% BFA in total are covered by the service areas of the existing urban parks. More urban park resources are located in the relatively developed districts and people in residential areas have more possibilities to access urban parks within a 10-min walking distance than people in workplace areas. Nonetheless, urban park resources are deficient around many residential areas which are the alternative places for new urban parks in future urban planning. However, there is a deficiency of the methods that it is hard to acquire the exact population scales in different areas according to BFA especially in workplace areas, which may make urban planners a bit confused since they get used to using population in their field.

## 2. Study Area and Data Sources

### 2.1. Study Area

**Figure 1:**
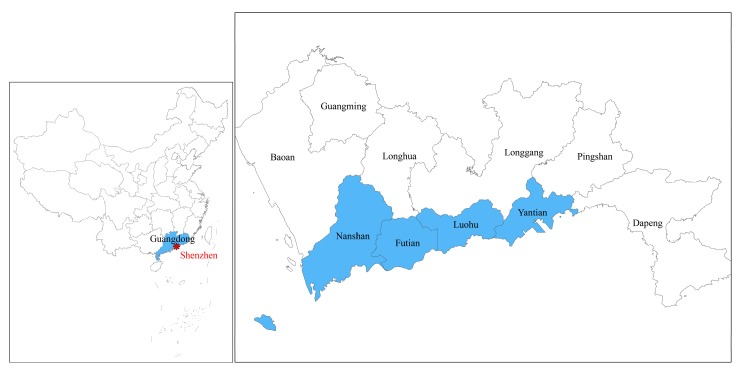
Study area: Shenzhen in Guangdong Province of China and its ten districts (the four blue-colored districts are the core districts).

Shenzhen is about 1997 km^2^ in size and its districts and administrative structures have been adjusted several times to balance the regional development, from an economic special region composed of four core districts (Luohu, Futian, Nanshan and Yantian) in 1980 to a megacity combining six extra districts (Baoan, Longhua, Longgang, Pinshan, Dapeng, Guangming) in 2010 (Figure 1).

Shenzhen has total 911 parks, including forest parks, city parks and community parks (http://www.szum.gov.cn/). These three kinds of parks are designed for different service capabilities. Generally forest parks and country parks are built at the areas with special natural landscapes such as mountains and waterfronts, many of which are a bit far from city centers and good for hiking or excursions during weekends or holidays [23,24]. City parks are designed for daily use, with larger sizes and better facilities than community parks with relatively limited spaces and facilities [23,24]. Therefore, city parks and community parks have more benefits than forest parks for people’s daily lives since people prefer parks in closer proximity to their homes during weekdays [2]. This study focused on the provision of city parks and community parks, and considering the differences between the two kinds of parks their service capacities were differentiated. In order to save the wording, the term “urban park” is used as a generic term for city park and community park in this paper when the both are referred to together.

### 2.2. Data Sources and Preprocessing

#### 2.2.1. Data Sources

This study applied several datasets, socio-economic dataset (including land area, Gross Domestic Product (GDP) and population in 2014), green-land survey dataset, building survey dataset and road network dataset of Shenzhen. The socio-economic dataset acquired from Shenzhen economic and social development statistics bulletin released by the Shenzhen Statistics Bureau in 2015 [20], was used to describe the economic context of each district in Shenzhen. The green-land and building survey datasets, derived from the datasets created by a survey of buildings and green spaces of Shenzhen in 2014, provided by the Urban Planning, Land and Resources Commission of Shenzhen Municipality. From the green-land survey dataset, the spatial locations and attribute of urban parks were extracted to acquire the spatial distribution of urban parks. The building survey dataset provides the spatial distribution of buildings and their attributes (e.g., floor area and usage) as well. The two survey datasets were also used to explore the potential service load of urban parks and the areas lacking urban park services. The road network dataset was used to create the service areas of urban parks taking into account spatial accessibility along the road connections from buildings to urban parks.

#### 2.2.2. Socio-Economic Contexts of Districts

**Figure 2:**
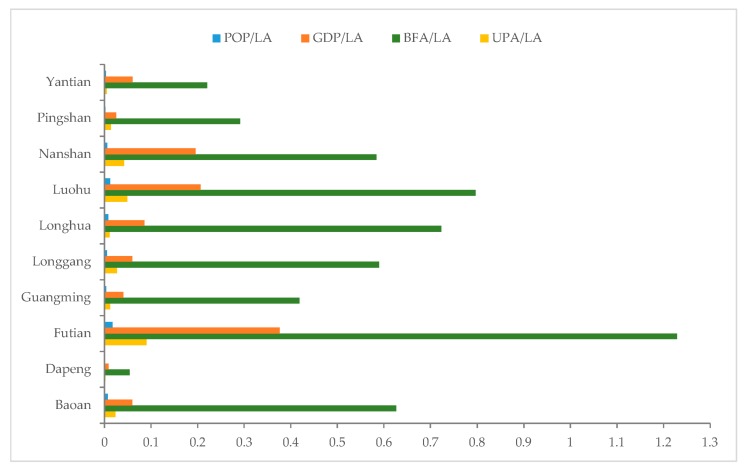
Socio-economic contexts of districts shown by ratio of (GDP), population (POP), building floor area (BFA) and urban parks area (UPA) to land area (LA) of each district.

The basic socio-economic contexts and urban parks of the ten districts in Shenzhen are illustrated in Figure 2 with the ratio of population (POP), GDP, BFA and urban parks area (UPA) of each district to their corresponding land area (LA). According to GDP, the economic context of Futian is the best and followed by Luohu and Nanshan. These three districts belong to the core districts and have been developed much better than the other districts. Especially for Futian, the central business district (CBD) is located there with the highest building density and population density and the most urban park resources. Longhua, Baoan and Longgang are at the second level of the economic contexts with relatively high building densities and urban park resources. The other districts have relatively lower performances in these items.

#### 2.2.3. Reasonability of Using Distribution of Buildings

**Figure 3:**
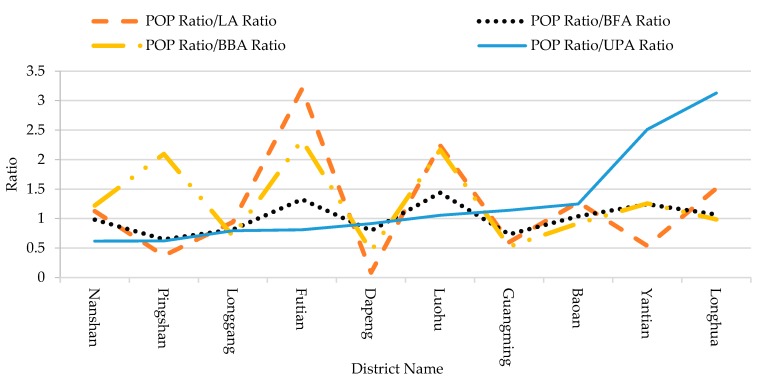
Ratio curves of the ratio of population (POP) to the ratios of land area (LA), building floor area (BFA), building base area (BBA) and urban park area (UPA).

Generally, an urban park is designed to provide effective services to people in a limited surrounding area, called as service area of the park which is varied with its size and category (see Section 3.2). BFA is related to the scale of population accumulated at cubical spaces in buildings on a piece of land. Therefore, it is benefit to estimate the real service capabilities of urban parks by measuring how much BFA is located within the service areas of urban parks. Beside the reasons using distribution of BFA to simulate distribution of population described in Introduction, a further verification was done by using the curves in Figure 3 and correlation analysis in Table 1 to prove the reasonability of such a simulation.

The ratio of population (POP Ratio) of each district was divided by the corresponding ratio of land area (LA Ratio), ratio of building floor area (BFA Ratio), ratio of building base area (BBA Ratio) and ratio of urban park area (UPA Ratio), respectively. The names of the districts along the *x*-axis is listed in ascending order of the value of POP Ratio/UPA Ratio. The dot-line curve expressing POP ratio/BFA Ratio fluctuates a little around 1 (0.3~1.4), while the other curves fluctuates a lot (0~3.2 for LA, 0.5~2.3 for BBA). Such results indicate that the distribution of population is proportionally similar to the distribution of BFA in all districts, which positively verify in a degree the reasonability and reliability of the above simulation.

The correlation analysis among POP, LA, UPA, BFA and BBA shows that the strongest correlation with POP is BFA (0.962) and the strongest correlation with UPA is BFA (0.854) (Table 1). This result further proves that the above simulation is reasonable and reliable.

#### 2.2.4. Classification of Building Usages 

The users are different for urban parks located in residential areas, commercial areas and industrial areas [19]. Generally, people at residential areas have higher possibilities to use urban parks than people at other areas in a day especially during weekend according to our observation. Therefore, this study generalized the usages of buildings into six categories based on the 2014 Shenzhen Urban Planning Standards and Guidelines [24], including residential, commercial, office, public infrastructure, industrial and other buildings (such as warehouses where no people stay often, or without usage information in the survey dataset of buildings).

In Shenzhen, there are two kinds of residential buildings, commercial residential buildings (CRB) and urban village residential buildings (UVRB). CRBs are built by real estate developers generally with their own gardens or public green spaces, while UVRBs are built by owners themselves in urban villages with little consideration of public spaces. Thus, naturally, people living in UVRBs more rely on public urban parks as entertainment spaces than people in CRBs. Such difference between the two kinds of residential buildings will be considered in evaluating the spatial provision of urban park distribution in the subsequent sections.

## 3. Methods

In this study, the spatial provision was explored by analyzing the spatial distribution of urban parks, the spatial coverage of their service areas and their potential service load. In addition, the places lacking urban parks were also identified based on the buildings outside the service areas of the existing urban parks.

### 3.1. Spatial Distribution of Urban Parks

The analysis of the spatial distribution of urban parks aimed to clarify the spatial association between urban parks and their potential users. Since it is reasonable to simulate the distribution of population with the distribution of BFA as described above, a building density map was made to describe the spatial distribution of BFA by setting BFA as a population field of the density tool in ArcGIS 10.1 (ESRI, Redlands, CA, USA). Then the spatial data of urban parks were overlapped on the map to display the spatial associations between urban parks and buildings. The result does not only consider the counts of buildings but also involves the volume of buildings with BFA. Therefore, the larger BFA will show the higher density if the counts of buildings are equal. The values of BFA-based building densities were graded into five classes with a natural breaks classification based on natural groupings inherent in the data first and then displayed on a map with graduated colors.

For commercial buildings, it is assumed that their population is proportional to floor area [19], and for residential buildings, their population is also estimated according to BFA per capita. On this density map, therefore, the higher density of buildings surrounding an urban park indicates the more potential users that the park may have. If there is no one urban park near a group of high-density buildings, urban park resources are scarce in this area. Therefore, this map can effectively display the spatial association between urban parks and their potential users.

Besides the above building density map, Index 1 (Equation (1)) was designed to further measure the spatial distribution of urban parks with the percentage of UPA to BFA:

Index 1 = UPA_d_/BFA_d_ (1)

where UPA**_d_** is the total area of urban parks and BFA_d_ is the total floor area of buildings in a district. The larger value of Index 1 means the more urban park resources possessed by a district.

This index measures the general scale of urban parks in each district based on the effective area suitable for people living or working. In some extant studies, the ratio of urban park area to land area of a community or planning unit is used to express the distribution of urban parks [12]. It is possible that this will fail to reflect the actual relationship between the distribution of urban parks and the distribution of population in some cases. For example, according to Figure 2, the LA of all districts but Futian is larger than the BFA. The ratio using LA may overestimate the scale of urban parks in Futian but may underestimate that in other districts. Especially for some districts (e.g., Dapeng and Yantian), there are a large pieces of lands which are not available for people to live or work (such as water bodies or mountains), so such a ratio also may produce relatively large biases, while, Index 1 (Equation (1)) only involves the area available for peoples staying in a district.

### 3.2. Spatial Coverage of Urban Park Services

Generally, an urban park is designed to provide effective services to people in a limited surrounding area, which means each park has an effective spatial service coverage. Hence, people who are inside spatial coverage of service of an urban park have higher possibility and can more easily use the park daily than people a bit farther away from the park [7]. Several distances were employed in literature to calculate the effective service areas of urban parks, such as 250, 300, 400, 800 and 1000 m [6,12,16,19]. Since this study was focused on daily services of urban parks within walking distance, two distances were applied, 5 min-walking distance (about 400 m) and 10 min-walking distance (about 800 m). The former one is a comfortable distance and the latter one is a little bit long, but still acceptable for most people.

Considering the differences in service capabilities and attractiveness between city parks and community parks, 400 m and 800 m were applied to calculate the service areas of community parks and city parks respectively to investigate the spatial coverage of their service areas. The service areas were generated based on the road networks of Shenzhen and only roads available for pedestrian were involved in the road networks. The network analyst tools of ArcGIS 10.1 were used to generate a service area of each urban park based on such road network. Therefore, if a building is located within the service area of a city park, people in this building can walk to this park within 10 min. For a community park, the time is 5 min.

When several urban parks are close to each other, their service areas are possibly overlapped. If a building is covered by the service areas of several urban parks, people in this building have more than one urban park available nearby. On the other hand, if a building is not covered by any park, people in this building have to walk a longer distance than 800 m to an urban park. Therefore, the times of buildings covered by the service areas of urban parks can discover the differences of the spatial coverage of urban park services.

In addition, Index 2 (Equation (2)) was designed to calculate the proportion of BFA covered by the service areas of urban parks in a district, and it reflects the potential scale of effective services of urban parks:

Index 2 = BFA_s_/BFA_d_ (2)

where BFA_s_ refers to the total floor area of all buildings within the service areas of all urban parks and BFA_d_ is the total floor area of all buildings in a district. The larger value of Index 2 means the more BFA covered by the service areas of urban parks, which indicates in a certain degree that the more people can access urban parks in acceptable walking distance. For city parks, BFA_s_ is the floor area of buildings in 800 m service areas, and for community parks it is the floor area of buildings in 400 m service areas.

As described at Section 2.2.4, the people in UVRBs more rely on urban parks as entertainment places than people in CRBs. So what is the provision of urban parks for people in the two kinds of residential buildings in Shenzhen? Thus, Index 3 (Equation (3)) was designed to distinguish the situations of CRBs and UVRBs covered by the service areas of urban parks to answer this question.

Index 3 = (BFA_CRB-s_/BFA_UVRB-s_)/(BFA_CRB-d_/BFA_UVRB-d_) − 1 (3)

where BFA_CRB-s_ and BFA_UVRB-s_ are the floor area of CRBs and UVRBs within the service areas of urban parks, respectively, BFA_CRB-d_ and BFA_UVRB-d_ are the corresponding floor area of the two kinds of residential buildings in a district.

If the value of Index 3 is close to 0, it means that the proportion of CRBs and UVRBs located in the service areas of urban parks are almost equal to the corresponding proportion at the district level. Therefore, the people living in the two kinds of buildings have equal access to nearby urban parks. If the index value is smaller than 0, the proportion of UVRBs is larger than that of CRBs in the service areas, which indicates that the urban parks may provide services to more people in UVRBs.

In addition, in order to identify the spatial prevision of urban parks at workplace areas, this study compared the proportions of floor area of different building types within the service areas with their corresponding proportions at the district level. If there is no big differences between the two sets of the proportions, the distributions of urban parks are somehow balanced surrounding the different types of buildings.

### 3.3. Potential Service Load of Urban Parks

Generally, urban parks are designed with a certain limitation of service capacity. If the service load of an urban park is larger than its service capacities, the quality and effect of service would be decreased. Index 4 (Equation (4)) was designed to estimate the potential service load from residential buildings in the service areas of urban parks in each district:

Index 4 = (BFA_s_/BFA_p_)/(UPA_d_/UPA_p_) (4)

where BFA_p_ and UPA_p_ are BFA per capita and UPA per capita, respectively, BFA_s_ is the total floor area of all residential buildings covered by the service areas of urban parks, and UPA_d_ is the total area of all urban parks in a district. BFA_s_/BFA_p_ was used to estimate the population living in the residential buildings covered by the service areas of urban parks, and UPA_d_/UPA_p_ was used to estimate the population which would be held in urban parks of a district according to the UPA per capita at the city level. The larger value of Index 4 indicates the larger service load of urban parks in a district.

### 3.4. Lack of Urban Park Services

Since the service areas of city parks and community parks are calculated within 800 m distance (i.e., 10-min walking distance) and 400 m distance (i.e., 5-min walking distance) along available roads, it is reasonable to suppose that people in buildings not covered by the service area of urban parks cannot reach urban parks by walking for 10 min. Index 2 not only describes the spatial coverage of the service areas but also the spatial scale of buildings lacking urban park services. In order to identify the explicit places lacking urban parks, the buildings outside the service areas were extracted to create a new BFA-based building density map. The high-density areas are the right places for planning new urban parks in future.

Since people in residential areas generally have higher needs for urban parks than people in working areas, it is more significant to make clear the lack of urban parks in residential areas in order to determine the right places for developing new urban parks in the near future. Therefore, Index 5 (Equation (5)) was designed to estimate how many urban park resources would be needed for residents in residential buildings outside the service areas of the existing urban parks:

Index 5 = (BFA_os_/BFA_p_)/(UPA_d_/UPA_p_) (5)

where BFA_os_ is the building floor area outside the service area of urban parks, and the other variables have the same meanings as the ones of Index 4. The larger value of Index 5 indicates that the scale of urban parks needed for residents outside the service areas are even larger than the existing urban parks.

## 4. Results

### 4.1. Spatial Distribution of Urban Parks

**Figure 4:**
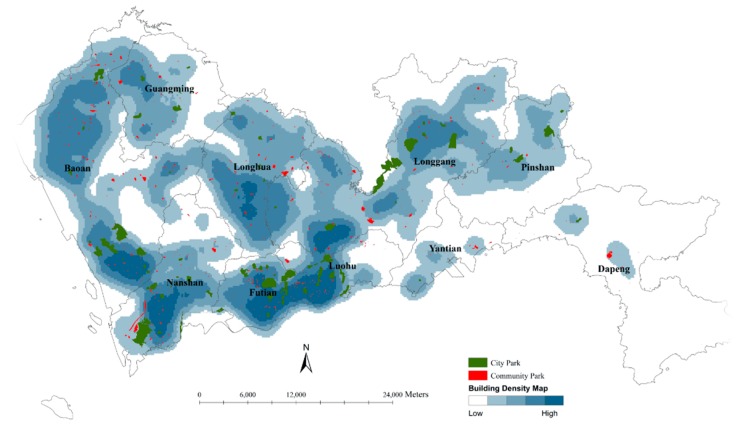
Building density map based on BFA (darker blue areas refer to higher-density areas and blank areas refer to no building or very fewer buildings) and spatial locations of city parks (green color) and community parks (red color).

According to the methods described in Section 3.1, a BFA-based building density map was made and overlapped by city parks (green color) and community parks (red color) (Figure 4). Visually, the building densities at Nanshan, Futian, Luohu, the south part of Baoan and Longgang are at the first high level with several city parks and some community parks nearby. The building densities at the north part of Baoan and the middle of Longhua are at the second level with many community parks nearby. At Yantian, Dapeng, and Pingshan, the densities of buildings are relatively lower and the resources of urban parks are also relatively limited.

### 4.2. Results of Proposed Indices

Table 2 lists the results of the five indices designed in Section 3. According to Index 1, Futian, Nanshan and Luohu have better urban park resources than the other districts, which are consistent with their socio-economic contexts shown in Figure 2. Index 2 indicates that Futian and Luohu have larger spatial coverage of urban park services because there are more than 70% BFA covered by the service areas of the urban parks. Such results attribute to the high densities of buildings in the two districts. Nanshan and Yantian also have good performance with more than 50% BFA within the service areas. In the other districts, fewer than 35% BFA are located in the service areas. Such results reveal the clear differences in the provisions of urban park resources between the four core districts (shown in Figure 1) and the other districts.

The results of Index 3 show that Pingshan is very special. According to the value of Index 2, the BFA within the service areas of the urban parks in Pingshan is 22.2%, the smallest one, however, the value of Index 3 is 1.3, the largest one. Only 23% out of those buildings inside the service areas are UVRBs, but the proportion of the total UVRBs in Pingshan is 33%. It indicates that in Pingshan people living in UVRBs have fewer urban park resources available within a 10-min walking distance than people in CRBs. In general, therefore, the provision of urban park resources is not balanced for people in UVRBs and CRBs in Pingshan, while, in several districts (e.g., Baoan, Dapeng, Longhua and Yantian) with negative values of Index 3, people in UVRBs have more urban park resources than people in CRBs.

Every year, a statistical yearbook is released in Shenzhen and BFA per capital residential building and UPA per capita are included. These two values in the Shenzhen Statistic Yearbook for 2015 [20] were used as BFA_p_ (i.e., 21.1 m^2^/person) and UPA_p_ (i.e., 16.8 m^2^/person) in Index 4 to estimate the service load of urban parks. The results of Index 4 show that the residential population in the service areas are more than two times the population able to be held in the urban parks in all of the districts, especially Yantian, where it is 14.34 times. Although the urban park resources available in Yantian are the last but one according to Index 1 (2.22%), the proportion of BFA (Index 2) inside the service area of the urban parks is almost 60% of the whole district. It means that the density of population is very high inside the service areas and thus it is bound to result in a big service load for the existing urban parks in Yantian. Meanwhile, Pingshan is an opposite case with the smallest value of Index 4 (2.02), a middle level of Index 1 (4.69%) and the smallest value of Index 2 (22.2%), which indicates that the density of population is not high enough to provide a good volume of potential users to the existing urban parks in Pingshan. In addition, the potential service load in Luohu and Longhua are also large (Index 4 > 7).

Index 5 estimates the necessary of new urban parks for residential population outside the service areas at the present. Pingshan and Longhua lack urban park resources most according to their values of Index 5 (3.53 and 3.52). As analyzed above, the existing urban park resources may not have a good use in Pingshan because of fewer potential users, and in Longhua the urban park resources are very limited according to Index 1 (1.53%). While, for the four core districts, the values of Index 5 are smaller than 1, which means that the scales of new urban park resources are smaller than the scales of the existing urban park resources.

### 4.3. Spatial Distribution of City Parks and Community Parks

**Figure 5:**
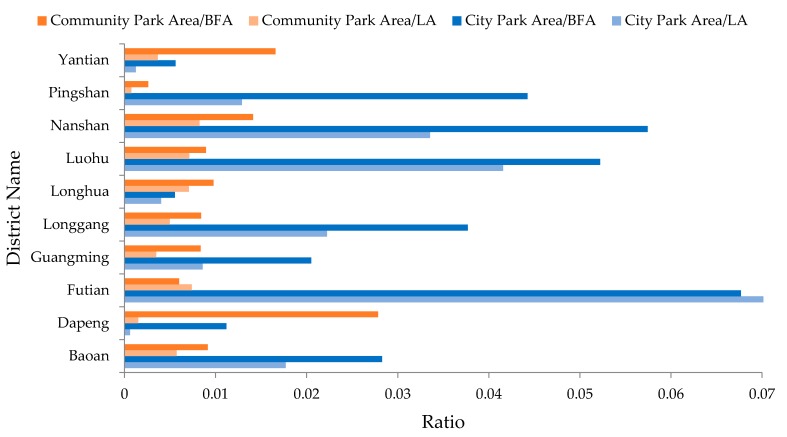
Spatial distribution of city parks and community parks based on building floor area (BFA) and land area (LA).

In order to describe the details of spatial distribution of city parks and community parks, Index 1 was calculated for city parks and community parks respectively. In addition, the ratio of community parks to BFA and the ratio of city parks to LA were calculated to reveal the different results of measuring the spatial distribution of the two kinds of parks with BFA and LA as described in Section 3.1 (Figure 5). The larger ratio means the more park resources possessed by a district. For all of the districts but Futian, the ratios to BFA are clearly larger than the ratios to LA. It proves that the ratio to LA may underestimate the spatial distribution of urban parks in these districts, but in Futian, it may overestimate.

Most of the districts but Yantian, Longhua and Dapeng have more city parks than community parks. According to Figure 2, the general UPA is very small in the three districts. The socio-economic context of Dapeng is the worst one, while GDP of Longhua is quite low according to its BFA proportion. Therefore, the economic situation may be the key reasons of the limited city parks in the two districts. Yantian is an industrial district with fewer population but its existing urban parks have very good potential users as described in Section 4.2. It will be discussed further in Section 4.4.

### 4.4. Spatial Coverage of Urban Park Services

**Figure 6:**
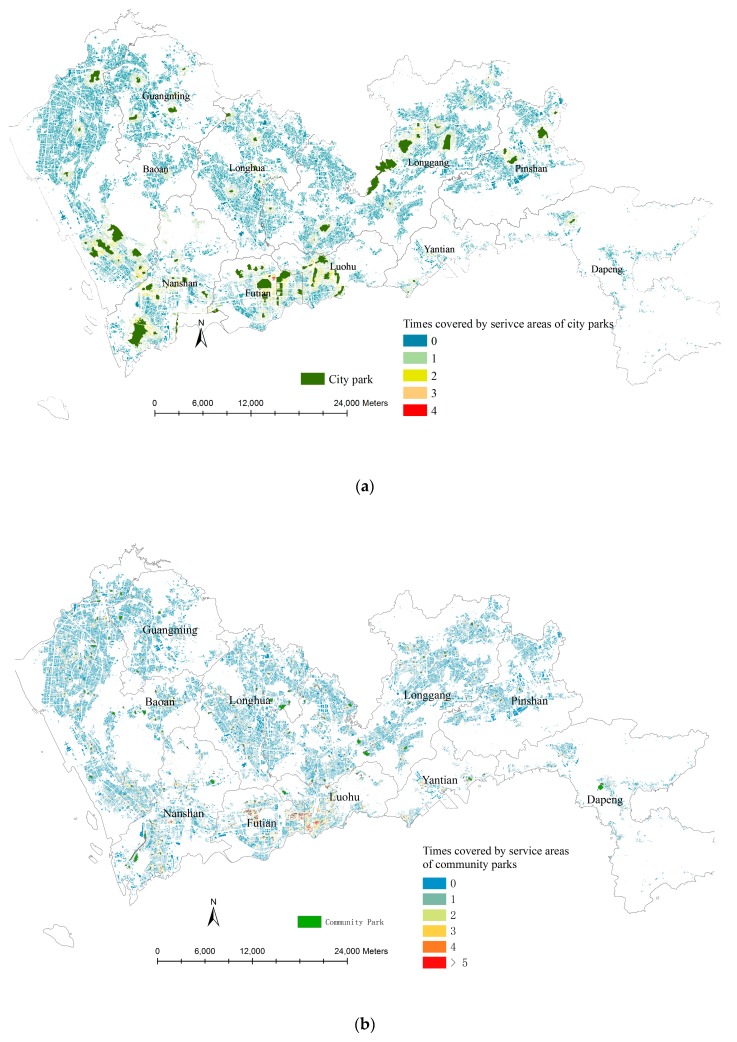
Times covered by service areas of city parks (**a**) and community parks (**b**) (Times = 0 meaning no city park in 10-min walking distance and no community park in 5-min walking distance, Times = 4 meaning there are 4 parks in 5-min walking distance, Times > 5 meaning more than 5 community parks in 5-min walking distance).

Besides Index 2, the times of buildings covered by the 800 m service areas of the city parks and the 400 m service areas of the community parks were counted (Figure 6). Some buildings are covered by the service areas of four city parks in Futian (times = 4, red color in Figure 6a), and some are covered by the service areas of more than five community parks in Futian, Luohu and Nanshan (times > 5, read color, Figure 6b). Meanwhile, a large amount of buildings (about 65% buildings) are located outside the service areas of the existing city parks and community parks (times = 0, blue color, Figure 6a,b).

Both figures reveal the imbalance of the spatial distribution of urban parks. People in some places have more options of urban parks but some have none within an acceptable walking distance. In addition, the total spatial coverage of the community park services are wider than that of the city parks since the densities of the community parks are larger than that of the city parks.

As described Section 3.2, this study investigated the provision of urban parks in workplace areas, which were differentiated into six kinds of working places according to the type of usage of the buildings. Table 3 illustrates the proportions of BFA of the six building types in each district and the corresponding proportions in the service areas of the urban parks. The proportions of the residential buildings are the largest in both the districts and the service areas, which indicates most of potential users of the urban parks are residents in all districts. Further, the proportions of the residential buildings in the service areas of Baoan, Guangming, Longgang and Yantian are even 10% larger than the corresponding proportions in the district. Such results indicate that the provisions of the urban parks are more located near the residential buildings in these districts.

Besides the residential areas, some urban parks are located at the official and commercial areas Futian, Luohu and Nanshan according to the BFA proportions of the office buildings (more than 10%) and the commercial buildings (close to 10%), and the proportions in the service areas of the urban parks are almost same as the corresponding proportions in the district level. It indicates that the provision of the urban parks nearby the two kinds of buildings are reasonable. The proportions of the industrial buildings are over 20% in the district of Baoan, Guangming and Longgang, however, the corresponding proportions in the service areas are clearly smaller. Such results reveal that the provisions of urban parks are shortage surrounding the industrial buildings in the three districts.

### 4.5. Lack of Urban Park Services

**Figure 7:**
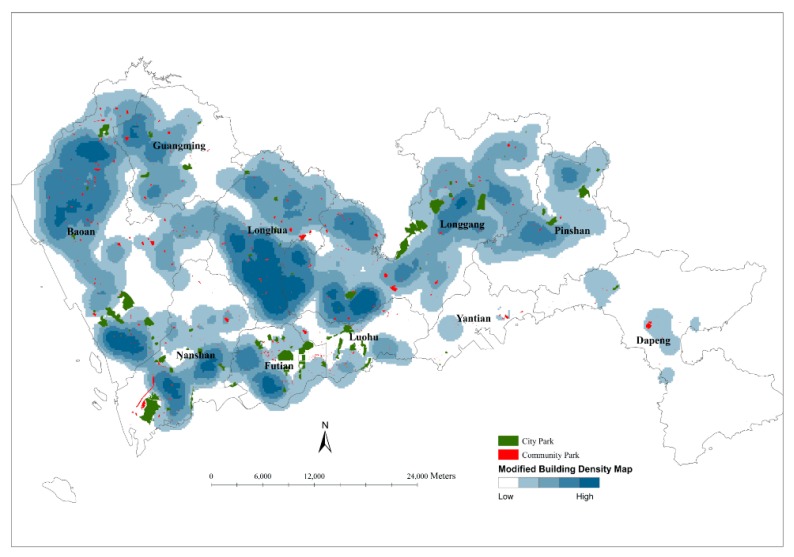
BFA-based density maps of the buildings outside the service areas of the urban parks (darker blue areas refer to higher-density areas and blank areas refer to no building or very fewer buildings).

As described above, in total there are about 65% buildings located outside the service areas of the existing urban parks. In Longhua, Longgang and Baoan, about 80%, 70% and 65% BFA are outside the service areas of the existing urban parks, respectively. In order to explore the concrete areas lacking urban park resources, a building density map was made based on the BFA of buildings outside the service areas (Figure 7). Clearly, the densities are very high at the south part of Longhua, Longgang and Baoan. Therefore, urban parks are very deficient in these areas.

**Figure 8:**
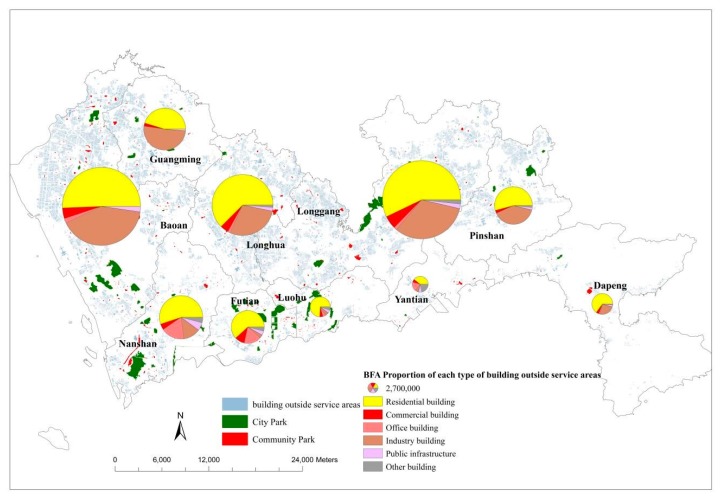
BFA proportion of each type of the buildings outside the service areas of the existing urban parks.

In addition, a cartogram map was made to investigate more in-depth the shortage of urban park resources in each district (Figure 8). All of the buildings located outside the service areas of the existing urban parks are displayed with gray parcels as a background, and each pie illustrates the detail information on the total BFA (with radius of pie) of such buildings of one district and the proportions (with angles of sectors) of the different building types. The larger pie implies the more buildings in which people do not have urban parks nearby in 10-min working distance and the more urgency to develop new urban parks.

In general, Baoan, Longgang and Longhua are the top that districts that need to urgently develop new urban parks. In most of districts except Guangming and Yantian, the residential areas need more urban parks since the proportions of the residential buildings are more than 50%. Besides, the industrial areas also clearly lack urban parks in the districts, except the four core districts with high proportions of industrial buildings (30–45%). In Futian and Nanshan, more urban parks are needed in the official areas since the proportions of office buildings are relatively large.

## 5. Discussion

This study evaluated the spatial provisions of urban parks based on their association with buildings in Shenzhen of China. The fundamental idea is that the spatial distribution of buildings can provide the exact locations where people live or work and using these locations one can precisely measure the accessibility to urban parks along the available roads. Further, it can estimate whether people in these buildings can reach urban parks by walking 10 min. Moreover, this study identified whether there were urban park resources located nearby residential areas and working places according to the types of buildings. Two kinds of residential buildings, CRB and UVRB, were differentiated since the dependence on public urban parks was different for people living in the two kinds of buildings. Based on the distribution of buildings, this study designed five indices to investigate the spatial provision of urban parks from various views and the results are also helpful to identify the potential places for planning new urban parks in the future.

**Figure 9:**
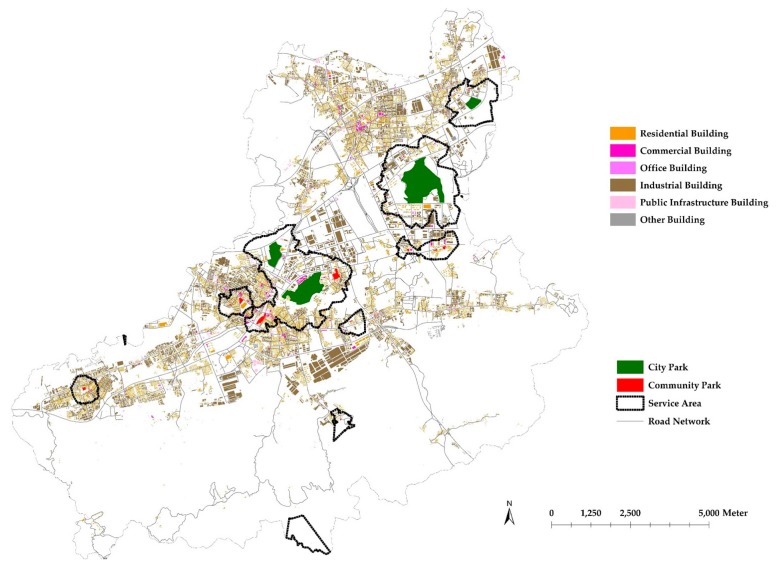
Distribution of urban parks and the coverage of their service areas in Pingshan district.

According to the analysis of the five indices, Pingshan is very special on the provision of the urban parks. Figure 9 illustrates the details of the spatial distribution of the urban parks in Pingshan and the spatial coverage of their service areas. The densities of the buildings are not high in the service areas of the four city parks. Totally, there are only 18% buildings with 24% BFA located in the service areas of the city parks and community parks. The minimum altitude difference of the four parks is larger than 20 m and the maximum difference is larger than 100 m. Thus, these four parks are mountain parks and their surrounding spaces would be limited for buildings, which may be the reason why these parks have no good potential users. At the north part of Pingshan, there are a large amount of residential, commercial and industrial buildings without any nearby urban parks. This area will be the right place for new urban parks in the future.

Oh and Jeong [19] used land area to calculate the service area ratio to evaluate the efficiency of parks, but according to our study case, using land area possibly misestimates the service capability of parks. If a study area has a large area of empty spaces or forest spaces like Yantian and Pingshan, using land area may underestimate the service capability of urban parks, while if the building density is very high in a study area, like in Futian, using land area may overestimate that (Figure 5). Oh and Jeong [19] applied BFA to evaluate the efficiency of parks in business/commercial areas which are different from residential areas. They take BFA within the service areas of parks as a substitute indicator of the number of park users in business/commercial areas, because it assumes that the population is proportional to the BFA of business/commercial areas. The method may be questionable if the provisions of urban parks are compared between business/commercial areas and residential areas since the indicators are calculated with the different methods.

Tan and Samsudin [12] also applied spatial coverage (or accessibility) of park services as an indicator to evaluate park provision, but the index which they applied is the ratio of land area within service areas of parks to the total land area of a planning unit. Such an index cannot reflect the real spatial coverage of urban park serivces when a planning unit is full of high-density buildings or has a large area of land not available for people to live or work. Thus the results of such an index may wrongly measure the spatial coverage of urban park services. In our study, land area was replaced with BFA in Index 2 to avoid the above problem.

## 6. Conclusions

This study proposed a solution for the evaluation of spatial provisions of urban parks by using the distribution of buildings in Shenzhen and differentiated the service capabilities of city parks and community parks. The study demonstrated that the spatial association between buildings and urban parks can investigate clearly the service capacities of the existing urban parks and discover the areas lacking urban parks as well. The findings in this study will provide helpful information for improving urban park planning. However, this study did not investigate the actual usages of urban parks and preferences, and in future the usage information will be incorporated in this study area.

## Figures and Tables

**Table 1:** A correlation analysis among POP, LA, UPA, BFA and BBA.

Correlation Sig.		LA	POP	GDP	UPA	BFA	BBA
LA	Pearson Correlation	1	0.587 *	0.150	0.536	0.719 **	0.799 **
	Sig. (1-tailed)		0.037	0.339	0.055	0.010	0.003
POP	Pearson Correlation	0.587 *	1	0.680 *	0.839 **	0.962 **	0.900 **
	Sig. (1-tailed)	0.037		0.015	0.001	0.000	0.000
GDP	Pearson Correlation	0.150	0.680 *	1	0.838 **	0.604 *	0.447
	Sig. (1-tailed)	0.339	0.015		0.001	0.032	0.098
UPA	Pearson Correlation	0.536	0.839 **	0.838 **	1	0.854 **	0.748 **
	Sig. (1-tailed)	0.055	0.001	0.001		0.001	0.006
BFA	Pearson Correlation	0.719 **	0.962 **	0.604 *	0.854 **	1	0.969 **
	Sig. (1-tailed)	0.010	0.000	0.032	0.001		0.000
BBA	Pearson Correlation	0.799 **	0.900 **	0.447	0.748 **	0.969 **	1
	Sig. (1-tailed)	0.003	0.000	0.098	0.006	0.000	

* Correlation is significant at a significance level of 0.05 (1-tailed). ** Correlation is significant at a significance level of 0.01 (1-tailed).

**Table 2:** The results of the five indices.

District Name	Index 1	Index 2	Index 3	Index 4	Index 5
	UPA_d_/BFA_d_	BFA_s_/BFA_d_	(BFA_CRB-s_/BFA_UVRB-s_)/ (BFA_CRB-d_/BFA_UVRB-d_) − 1	(BFA_s_/BFA_p_)/ (UPA_d_/UPA_p_)	(BFA_os_/BFA_p_)/ (UPA_d_/UPA_p_)
Baoan	3.74%	34.77%	−0.16	5.1	1.37
Dapeng	3.90%	27.57%	−0.42	3.78	2.54
Futian	7.37%	70.38%	0.37	4.68	0.42
Guangming	2.89%	28.59%	0.17	4.8	1.83
Longgang	4.61%	29.22%	0.12	3.6	1.94
Longhua	1.53%	22.51%	−0.18	7.1	3.52
Luohu	6.12%	83.00%	0.17	7.4	0.22
Nanshan	7.15%	54.38%	0.07	3.86	0.72
Pingshan	4.69%	22.20%	1.30	2.02	3.53
Yantian	2.22%	59.56%	−0.07	14.34	0.40

**Table 3:** Proportions of BFA of six building types in the district and in the service areas.

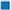	Residential building			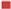	Commercial building			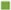	Public infrastructure		
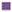	office building			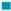	industrial building			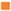	Other building		
BFA ratio in districts			BFA ratio in service areas			BFA ratio in districts			BFA ratio in service areas